# Syphilis

**Published:** 1916-10

**Authors:** 


**Syphilis.**

Intravenous injections of benzoate of mercury are condemned by L. Bizard and R. Klein (Ann. des Mal. Veneriennes, Dec. 1915) as accidents occur in nearly half the cases and the results are no better than from subcutaneous injections of the same drug and not so good as those from cyanid of mercury intravenously. They advocate as the best method, a daily intravenous injection of 1 c.g. of cyanid and a gluteal injection of 1-2 c.g. of benzoate. In milder or more susceptible cases, these methods are alternated.

In active military service, A. Bernheim (ibid. Oct. 1915) considers it sufficient to use benzoate or biniodid of mercury, 3 c.g. a day, with sulphur water as a drink or in baths, sodium arseniate and strychnine being also administered. Dental defects are treated (How about the danger to the dentist?) and dental hygiene inforced. After 21 days in the hospital, the soldier can be returned to active service without endangering his comrades or being liable to recurrences for a long time.

Mercurial poisoning, of scorbutic type, consecutive to treatment with grey oil. Ch. Audry, (Ann. de Derm. et de Syph., Oct. 1915) reports a case occurring in a man of 58 who contracted syphilis in 1888, and developed signs of tabes two or three years ago. After the sixth of a second series of injections of 10 drops of grey oil, the author saw the patient for the first time. He was very feeble and scarcely breathing. There was enormous tumefaction of the gums, intense nocturnal salivation, no ulceration, no albuminuria. The patient died in a few days. While rare, these cases may occur especially after the age of 50. While insoluble forms of mercury are valuable, they should be used with caution.

Serous apoplexy of salvarsan. Sauphar, (Soc. Med. des Hop., Dec. 21, 1915) states that most of the accidents imputed to salvarsan or neosalvarsan are of this type, occurring on the 3d or 4th day after the injection. With or without premonitory symptoms, the patient becomes comatose and dies in a few hours. Milan considers the pathology practically identical with that of cerebral crises due to nitrites, and hence advocates the use of adrenalin, intravenously, in large dose. Sauphar presents a case apparently saved from the almost inevitably fatal "serous apoplexy," by this method.

Incubation period, from medico-legal standpoint. G. Thibierge (Ann. de Derm. et de Syph., 1915) states that statistics show that the average duration of incubation is 25-30 days, that it is rarely less than 14-17 days and that it is difficult to fix the maximum except that the great majority of cases develop within 45 days, while the average period applies to only 40% of all cases.

Zoniform syphilides (the term should be either zonoid or cinguliform) Gaucher, Bizard and Bralez, (Ann. des Mal. Veneriennes, Oct. 1915) states that this particular manifestation of cutaneous syphilis was first described by Gaucher and Barbe in 1894. It occurs, according to both time and characteristics, as a tertiary lesion and assumes the 5 forms of the papulo-squamous type.

Bilateral palmar syphilide, of horny type, six years duration, rebellious to ordinary treatments and healed in 6 weeks by injections totalling 2.10 grams of arseno-benzol. Louis Queyrat (Soc. de Med. des Hop., Dec. 17, 1915).

Paternal heredity of syphilis. Gaucher (Ann. des Mal. Veneriennes, Jan. 1916 believes that, though denied by many, this form is demonstrated clinically and serologically, by the exclusion of maternal infection. Paternal heredity is perhaps less grave than maternal but it is much more frequent (sic). It occurs in three forms: 1. Virulent, which may occur even after several years' duration, the infant presenting generalized secondary syphilis—infantile syphilis properly so-called. This is the classic hereditary syphilis. 2. Remote consequences, parasyphilitic and dystrophic lesions. 3. Hereditary taints recognized only by careful study. The author emphasizes the necessity of anti-syphilitic treatment immediately before marriage, even when the man is apparently cured and the syphilitic infection remote in time.

Galyl. Arthur Foerster of London (Le Prog. Med., Jan. 5, 1916). This was synthetized by Mouneyrat, the discoverer of hectine. It is tetraoxy-diphospho-amino-diarsenobenzene, having in the molecule, 24 atoms of C, 22 of H, 8 of O, 4 of N, 2 of P, 4 of As, and containing by weight 35.3% of arsenic and 7.2% of phosphorus. It is a yellow powder, whose solution is no more difficult than for neosalvarsan. The best method of administration is by intravenous injection but intramuscular injections of oily solutions may also be employed, or otherwise as for neosalvarsan. A number of favorable cases are reported. Dyspnoea and partial syncope occurred after a dose of 50 c.c. but no unpleasant symptoms occurred after doses up to 40 c.g. As a routine, 30 c.g. for men and

20-25 c.g. for women are advised, repeating every 6-8 days. Mercurial treatment should follow. Petrini of Bucharest (Ann. des Mal. Veneriennes, April) also praises galyl and thinks that, on account of the dangers, salvarsan and neosalvarsan should be superseded.

French venereal statistics. Le Bull. Med., May 6, gives the following statistics for the first two months of the year:

| | Civil | inclusive of Class of 1917. | Military. | Total. |
|---|---|---|---|---|
| Blenorrhagias | 5487 | (1238) | 2326 | 7813 |
| Soft chancres | 824 | ( 122) | 138 | 962 |
| Syphilis | 1517 | ( 205) | 1470 | 2987 |
| Total | 7828 | (1505) | 3934 | 11762 |

Syphilis and wounds. L. Bory, (Reunion Med. de la lre Armee, Le Prog. Med., July 5) cites three trivial wounds of the leg, rapidly becoming gummatous ulcers and one case of gummata developing in an abdominal scar after a shrapnel wound. Antisepsis, curettage and irrigation were in vain till general antisyphilitic measures were instituted.

Hereditary syphilis and cyanosis. E. Jeanselme, (Presse Med., Dec. 2, 1915) reports the case of a syphilitic mother with 4 children. The oldest, a girl of 11, had typic signs of cyanosis, a boy of 6 manifested an abortive form of cyanosis. Both had other syphilitic lesions. A boy aged 7½ had neither cyanosis nor indications of syphilis but, like the other two, gave a positive Wassermann. The youngest child, a boy of 4 seems to have escaped infection altogether.

